# Evidence Synthesis and Mechanism Analysis of Quercetin Treatment for Atherosclerosis: A Preclinical Systematic Review and Meta-Analysis

**DOI:** 10.3390/ijms27010527

**Published:** 2026-01-04

**Authors:** Daiqian Chen, Jiawei Wang, Zhiguo Lei, Liping Qu, Wenjun Zou

**Affiliations:** School of Pharmacy, Chengdu University of Traditional Chinese Medicine, Chengdu 611137, Chinawangjiawei@stu.cdutcm.edu.cn (J.W.);

**Keywords:** quercetin, atherosclerosis, preclinical evidence, mechanism, meta-analysis

## Abstract

Atherosclerosis seriously endangers human health. Quercetin has drawn attention for its potential anti-atherosclerotic pharmacological effects. This study aimed to comprehensively assess quercetin’s effect and potential mechanism in treating atherosclerosis through a systematic review and meta-analysis. Preclinical studies published before 20 January 2025 were searched for in databases including PubMed, Embase, Web of Science, CNKI, Wanfang, and VIP. The CAMARADES list was used to assess the quality of the included studies. Stata 12 was applied for overall effect, sensitivity, subgroup, and publication bias analyses. Time–dose interval analyses were conducted to explore how quercetin dose and dosing cycle affect intervention effects. Finally, trial sequential analyses were performed using TSA 0.9 software. A total of 22 studies involving 421 animals were included, with a mean methodological quality score of 7.73/10. Meta-analysis showed that relative to the control group, quercetin reduced aortic plaque area, adjusted lipids (lowered TC, TG, and LDL-C and raised HDL-C), downregulated adhesion factors (e.g., VCAM-1) and pro-inflammatory factors (e.g., IL-1β and IL-6), upregulated anti-inflammatory factor IL-10 and antioxidant enzymes (SOD, CAT) while decreasing MDA content, and regulated atherosclerosis-related targets (e.g., LXRα, SIRT1, and mTOR). Subgroup analyses found model establishment time and quercetin administration time affected aortic lesion areas, TC, and TG. Time–dose analysis indicated quercetin had better ameliorative effects on atherosclerosis at 25–100 mg/kg with an 8–10-week intervention. Quercetin significantly improves atherosclerosis and inhibits its occurrence and progression through multiple pathways, such as regulating lipid metabolism, anti-inflammatory effects, and counteracting oxidative stress. Based on current evidence, quercetin is a potential therapeutic agent for treating atherosclerosis.

## 1. Introduction

Atherosclerosis (AS), which has become the leading cause of cardiovascular mortality worldwide, is a pathological disease characterised by fibrous proliferation of vessel walls, chronic inflammation, lipid accumulation, and immune disorders. As atherosclerotic plaques continue to develop, the fragile plaques are likely to rupture, leading to acute cardiovascular events including ischaemic stroke and myocardial infarction [1]. Atherosclerosis is characterised by high morbidity and mortality rates, with common causative factors including hyperlipidaemia, hypertension, smoking, and diabetes mellitus, but there may be a gradual restoration of the health of this population with changes in modern diet and exercise habits [2]. The main drugs used clinically for symptomatic treatment include lipid-lowering drugs (statins and niacin), antiplatelet and thrombolytic drugs (aspirin and urokinase), and anticoagulant drugs (warfarin) [3]. These drugs are effective in reducing atherosclerosis by lowering lipid levels, antiplatelet aggregation, and antithrombosis. However, the adverse effects of the drugs after long-term treatment have been widely documented; e.g., statins significantly reduce low-density lipoprotein (LDL) levels but may not adequately reduce LDL levels in all patients, and many patients are intolerant of statin therapy due to side effects (e.g., rare rhabdomyolysis). Long-term drug therapy can also cause side effects such as liver damage, gastrointestinal bleeding, muscle discomfort, and cardiac arrhythmias [4,5]. Therefore, there is an urgent need to develop an accurate and efficient treatment to reduce adverse effects, improve therapeutic efficacy, and, more importantly, increase patient compliance and improve prognosis.

Plant-derived polyphenols, such as flavonoids, have potential health benefits and no obvious side effects on humans [6]. Many fruits, vegetables, grains, nuts, and herbs are rich in flavonoids, which also exist in the leaves and flowers of a few plants [7]. Epidemiological studies have shown that foods rich in flavonoids can prevent some diseases, including metabolism-related diseases and cancer. The latest evidence shows that flavonoids have antioxidant, anti-inflammatory, analgesic, anti-cancer, anti-angiogenesis, anti-microbial, and antihypertensive properties [8,9,10]. Quercetin is a polyphenolic natural flavonoid abundant in almost all edible vegetables and fruits [11]. Moreover, bee products such as propolis, bee bread, and bee pollen are also rich in quercetin [12,13]. Relevant studies indicate that quercetin derived from these bee products similarly exhibits significant pharmacological activities, including anti-inflammatory and antioxidant effects, thereby further expanding the natural sources and application potential of quercetin [14,15]. Modern pharmacological studies have shown that quercetin possesses anti-inflammatory, antioxidant, anti-tumour, and cardioprotective properties [16]. Extensive investigational experiments using cultured cells and model animals have demonstrated that quercetin has great potential for use in the treatment of atherosclerosis [17]. Since 2010, a number of studies on using quercetin for the treatment of atherosclerosis have been reported, including several high-quality preclinical trials. In the present study, we performed a systematic evaluation and meta-analysis by pooling data from relevant animal studies to analyse the protective effects of quercetin and its mechanism of action in an animal model of atherosclerosis, providing evidence and theoretical references for subsequent clinical studies.

## 2. Methods

### 2.1. Programme and Registration

**Figure 1:**
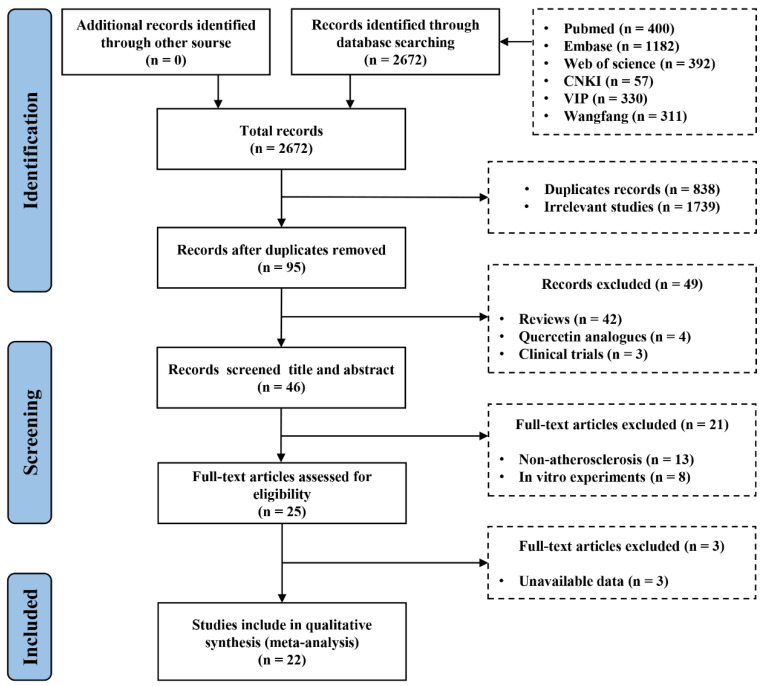
PRISMA flow diagram of the present study.

We strictly followed the Preferred Reporting Items for Systematic Reviews and Meta-Analyses (PRISMA) for the design and performance of systematic evaluations and meta-analyses. The PRISMA screening process is shown in Figure 1. The study protocol has been registered on the International Prospective Register of Systematic Reviews (PROSPERO) under the number CRD42024503334.

### 2.2. Search Strategy

In order to obtain more comprehensive results, three Chinese electronic databases and three English databases (PubMed, Embase, and Web of science) were searched via computer from inception to 20 January 2025. The Chinese databases were China National Knowledge Infrastructure (CNKI, https://www.cnki.net/, accessed on 20 January 2025), Wan Fang database (Wanfang, https://www.wanfangdata.com.cn/), and Chinese Scientific Journal Database (VIP, https://www.cnki.net/). Search entries included (“Quercetin” [MESH] OR “Quercitin” [MESH] OR “Que” [MESH]) AND (“Atherosclerosis” [MESH] OR “Atherosclerosis plaque” [MESH]). Endnote (a specialised software product used for searching and managing the literature) was used to identify and remove duplicate or inappropriate studies.

### 2.3. Study Selection and Inclusion Criteria

Inclusion criteria were established per the PICO framework: the population comprised animal models of atherosclerosis generated through validated methodologies; intervention required sustained quercetin monotherapy; control groups consisted of untreated atherosclerotic counterparts; and outcome assessment required evaluation of aortic lesion areas (quantitatively or qualitatively) to confirm atherosclerosis pathology, with the primary biochemical parameters being total cholesterol (TC), triglycerides (TGs), high-density lipoprotein (HDL-C), and low-density lipoprotein (LDL-C), while elucidation of quercetin’s underlying vasculoprotective mechanisms served as secondary outcomes.

Exclusion criteria encompassed non-atherosclerotic models; interventions involving non-quercetin compounds or combination therapies; control groups receiving active treatments; studies lacking aortic lesion assessment; non-primary research including reviews, case reports, conference abstracts, clinical trials, and in vitro studies; inaccessible full-text publications despite meeting inclusion criteria; duplicate datasets or publications; and studies failing to report sample sizes with associated dispersion metrics (standard deviation [SD] or standard error of mean [SEM]).

### 2.4. Data Extraction and Quality Assessment

Records from the literature were imported into EndNote X9 for deduplication. Dual independent screening was performed by two investigators using predetermined eligibility criteria, commencing with title/abstract assessment to exclude non-relevant publications followed by full-text evaluation of retained articles. The data extracted encompassed first author; publication year; language; experimental animal characteristics (species, sex, sample size, and body weight); anaesthetic protocols; pharmacological interventions (administration route, dosage, and treatment duration); and outcome measures with intergroup differences. For studies employing multiple quercetin doses, only the highest-dose cohort was included in meta-analysis. Graphically presented data were quantified using digital measurement tools, while missing standard deviations were calculated from reported standard errors using the Cochrane-recommended formula (SD = SEM × $\sqrt{n}$). Study quality was independently appraised with the CAMARADES 10-item checklist, modified to incorporate anaesthetic selection criteria (i.e., agents not significantly confounding outcomes). Discrepancies in quality assessments were resolved through consensus adjudication.

### 2.5. Statistical Analysis

Statistical analyses were conducted using Stata 12.0. Continuous outcome variables were expressed as standardized mean differences (SMDs) with 95% confidence intervals. Heterogeneity was quantified using *I*^2^ statistics, with fixed-effects models applied when *I*^2^ < 50% or *p* > 0.05; otherwise, random-effects models were employed. Sensitivity analyses assessed result robustness, while stratified subgroup analyses investigated heterogeneity sources in aortic lesions, TC, TG, and LDL-C—parameters reported by most of the included studies—categorized by publication language, animal species/sex, modelling duration, and quercetin administration parameters (method, dosage, and duration). Publication bias was evaluated via Egger’s test. Dose–time response relationships for key parameters were visualized using Origin 2022. Trial sequential analysis (TSA 0.9) with α = 0.05 and 80% power verified the meta-analysis’s reliability for TC and LDL-C, wherein cumulative Z-curves crossing both required information size (RIS) and TSA boundaries indicated conclusive evidence without further trials.

## 3. Results

### 3.1. Study Identification

Via this retrieval strategy, 2672 pieces of data were retrieved. After the duplicate and irrelevant studies were eliminated, 95 articles remained. A total of 46 studies were included after literature reviews, clinical trials, quercetin analogues, etc., were further excluded. We excluded non-atherosclerotic models and in vitro studies through further screening by reading the full texts. Three studies were excluded because valid data could not be obtained. Finally, 22 studies met the qualification requirements for full-text evaluation and were included in this systematic review and meta-analysis [18,19,20,21,22,23,24,25,26,27,28,29,30,31,32,33,34,35,36,37,38,39]. The included studies were published between 2011 and 2023, indicating that quercetin’s protective effect on atherosclerosis and the mechanism of this effect have been a research hotspot in recent years.

### 3.2. Research Characteristics

**Figure 2:**
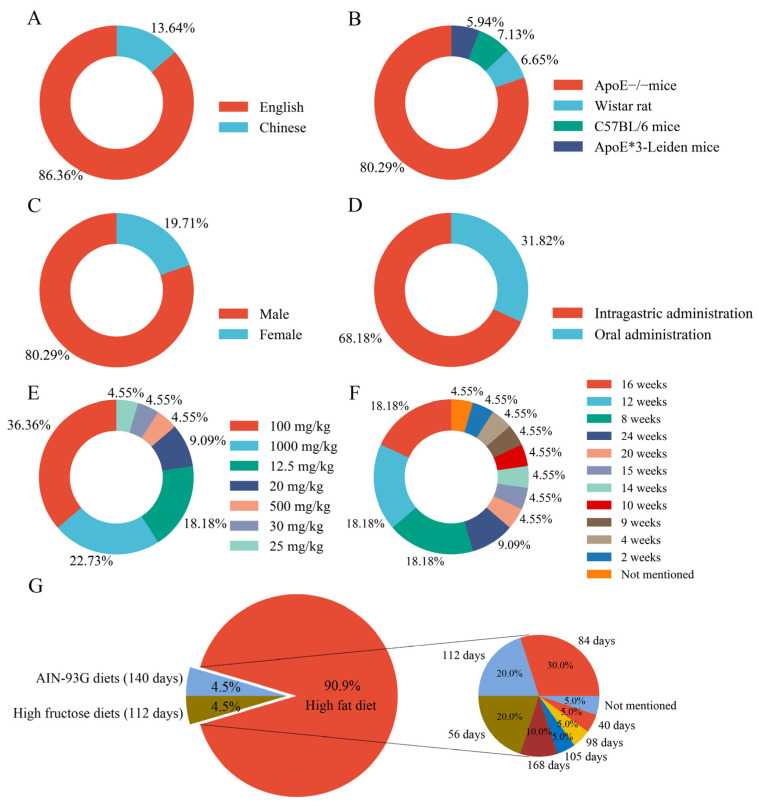
Characteristics of the included studies. (**A**) Language type; (**B**) animal species; (**C**) animal sex; (**D**) administration method of quercetin; (**E**) dosage of quercetin administered; (**F**) duration of quercetin administration; (**G**) modelling method. *Note:* ApoE*3-Leiden is a transgenic mouse model that simulates human lipoprotein metabolism characteristics by introducing a point mutation (a specific amino acid substitution) into the apolipoprotein E gene. It is frequently employed in research on atherosclerosis.

Table 1 summarizes the baseline characteristics of the 22 included studies, with quercetin intervention details presented in Table 2. English publications predominated (86.36%, 19/22) relative to the Chinese literature (13.64%, 3/22). The 421 experimental animals comprised apolipoprotein E-deficient (apoE^−^/^−^) mice (80.29%, 338/421), Wistar rats (6.65%, 28/421), C57BL/6 mice (7.13%, 30/421), and ApoE*3-Leiden mice (5.94%, 25/421). Male subjects constituted 80.29% (338/421) of the population, while females made up 19.71% (83/421). Quercetin administration occurred via oral gavage (68.18%, 15 studies) or drinking water (31.82%, 7 studies), with dosages primarily distributed at 100 mg/kg (36.36%), 1000 mg/kg (22.73%), and 12.5 mg/kg (18.18%). Treatment duration was predominantly 8–16 weeks (54.54% collectively), extending to 24 weeks (9.09%). In atherosclerosis modelling, high-fat diets were used (90.9%), with purified AIN-93G or high-carbohydrate regimens being employed for residual cases (Figure 2). Anaesthetic protocols included pentobarbital sodium (10 studies) and isoflurane (2 studies), while 9 studies omitted anaesthetic specifications. Primary-outcome reporting rates were as follows: aortic lesions (77.27%), total cholesterol (72.73%), triglycerides (81.82%), HDL-C (54.55%), and LDL-C (63.64%). Supplemental investigations encompassed inflammatory mediators (e.g., IL-6 and TNF-α), oxidative stress markers (MDA and SOD), and molecular targets, including NADPH and HO-1.

### 3.3. Quality Evaluation

**Figure 3:**
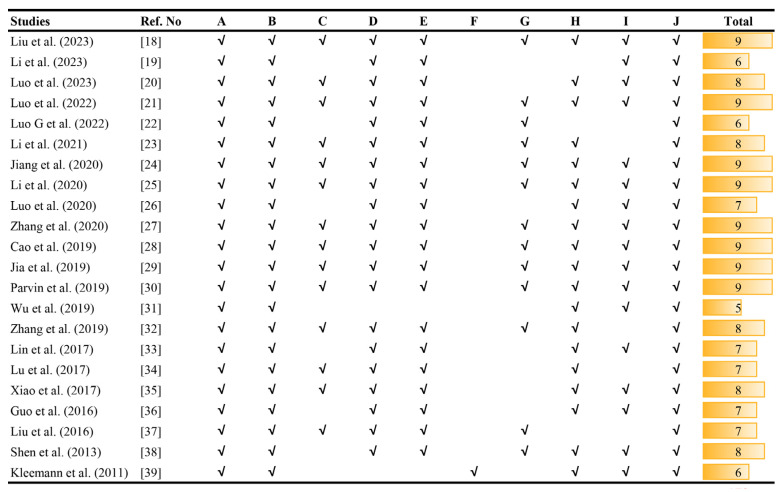
Risk of bias and quality scores of included studies. *Note:* A, Appropriate animal model; B, sample size calculation; C, control of temperature; D, random allocation to treatment or model; E, allocation concealment; F, blinded assessment of outcome; G, application of anaesthetics—indicating the method of anaesthesia—that do not significantly affect outcome indicators; H, compliance with animal welfare regulations; I, statement of potential conflicts of interest; J, peer-reviewed publication. *Note:* √ indicates compliance with the criteria for the revised CAMARADES checklist [18,19,20,21,22,23,24,25,26,27,28,29,30,31,32,33,34,35,36,37,38,39].

The methodological quality of the 22 included studies was appraised using the modified CAMARADES checklist, yielding scores ranging from 5 to 9 (mean = 7.73), collectively indicating robust study quality. All investigations satisfied the core criteria: appropriate atherosclerosis models were used, the sample size was justified, and the publications were peer-reviewed. Additional quality metrics revealed that 63.64% (14/22) implemented temperature control; 4.55% (1/22) utilized blinded outcome assessment; 59.09% (13/22) employed anaesthetic protocols without significant outcome confounding; 86.36% (19/22) complied with animal welfare regulations; and 77.27% (17/22) disclosed no conflicts of interest. Comprehensive bias assessment is detailed in Figure 3.

### 3.4. Effectiveness

#### 3.4.1. Primary Outcomes

**Figure 4:**
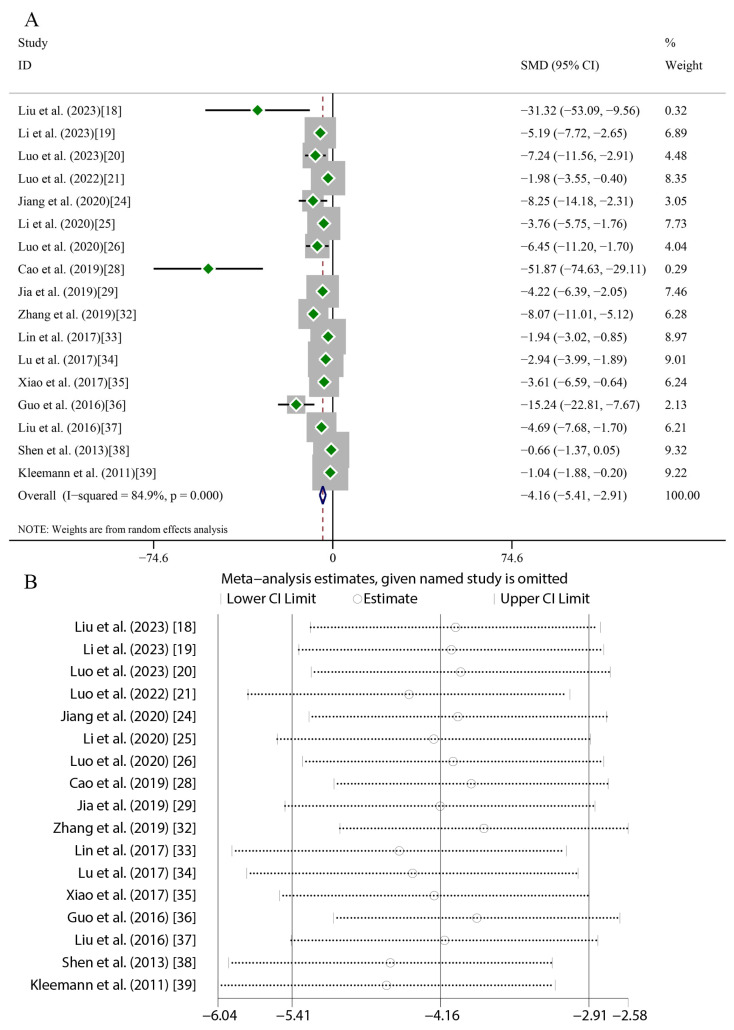
Forest plot (**A**) and sensitivity analysis (**B**) of aortic lesion areas [18,19,20,21,24,25,26,28,29,32,33,34,35,36,37,38,39].

**Figure 5:**
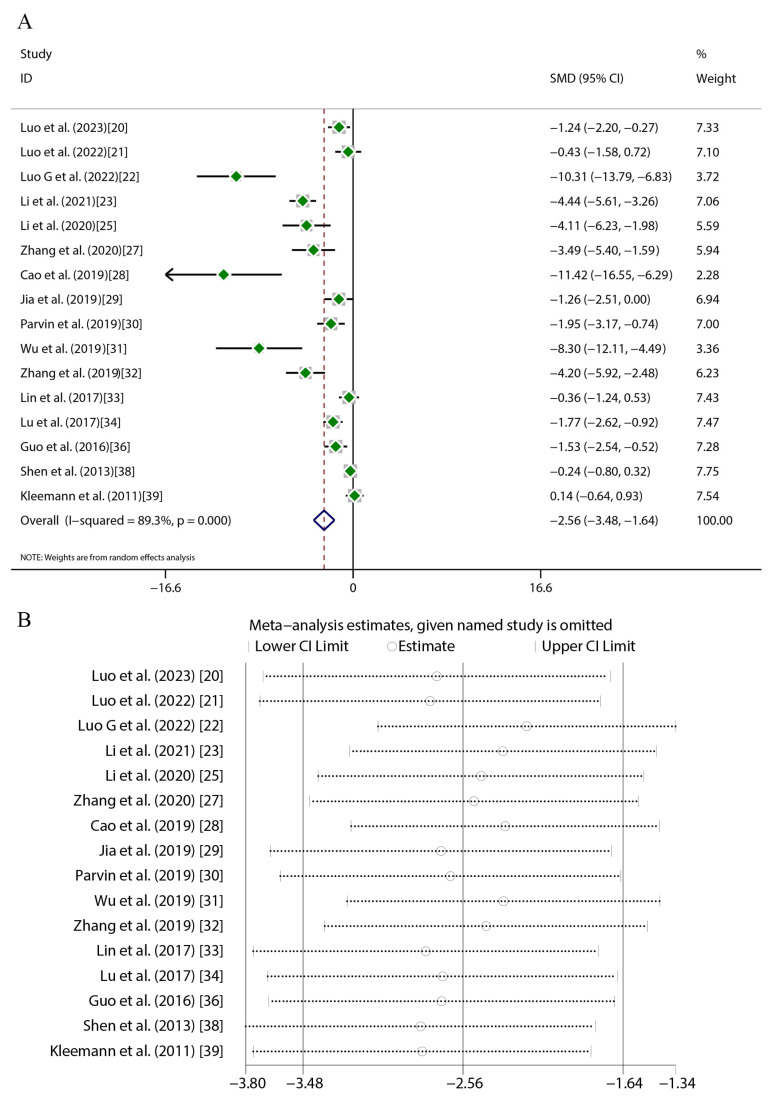
Forest plot (**A**) and sensitivity analysis (**B**) of TC levels [20,21,22,23,25,27,28,29,30,31,32,33,34,36,38,39].

**Figure 6:**
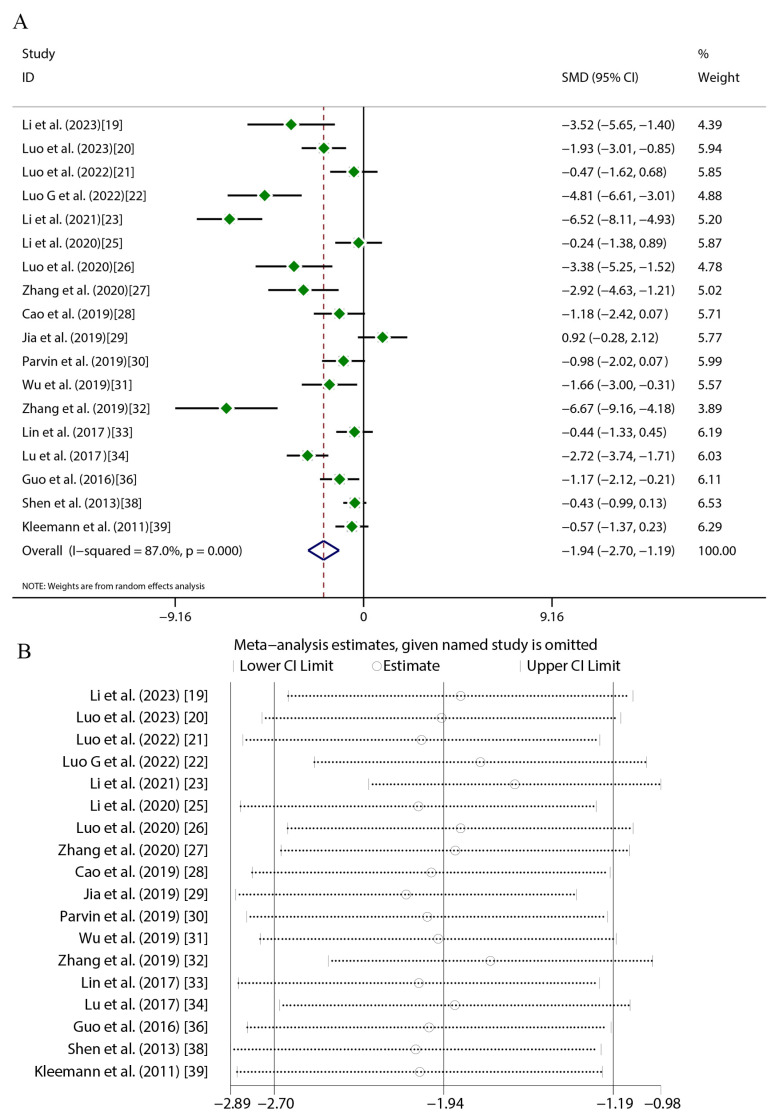
Forest plot (**A**) and sensitivity analysis (**B**) of TG levels [19,20,21,22,23,25,26,27,28,29,30,31,32,33,34,36,38,39].

**Figure 7:**
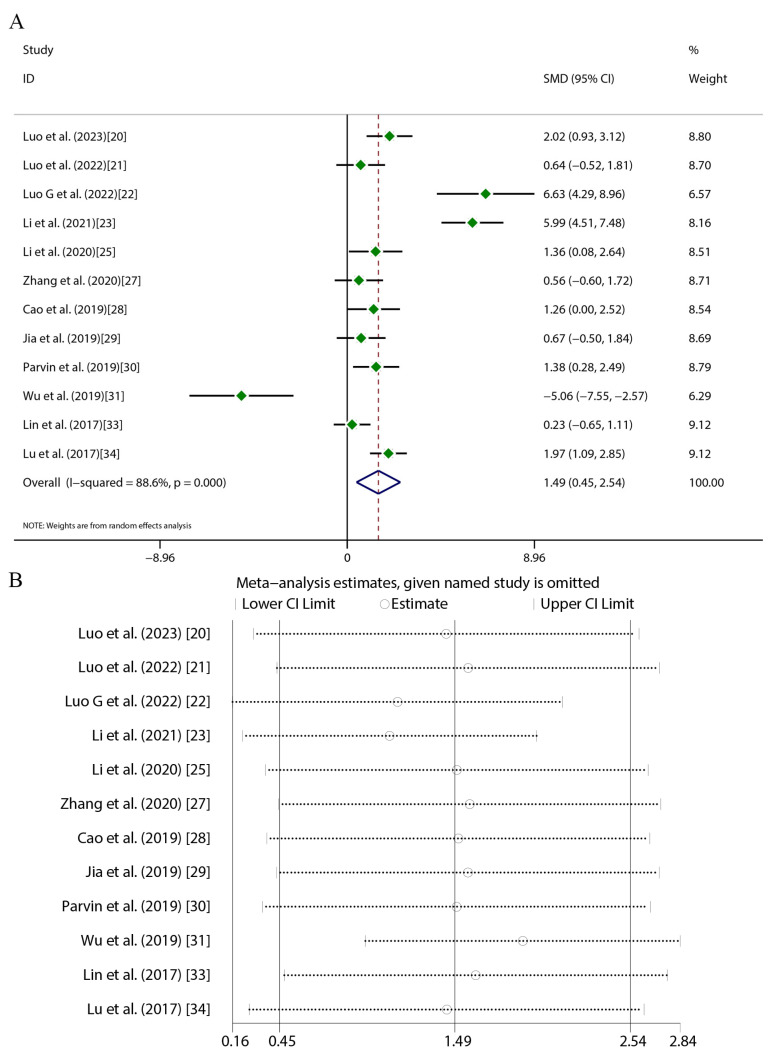
Forest plot (**A**) and sensitivity analysis (**B**) of HDL-C levels [20,21,22,23,25,27,28,29,30,31,33,34].

**Figure 8:**
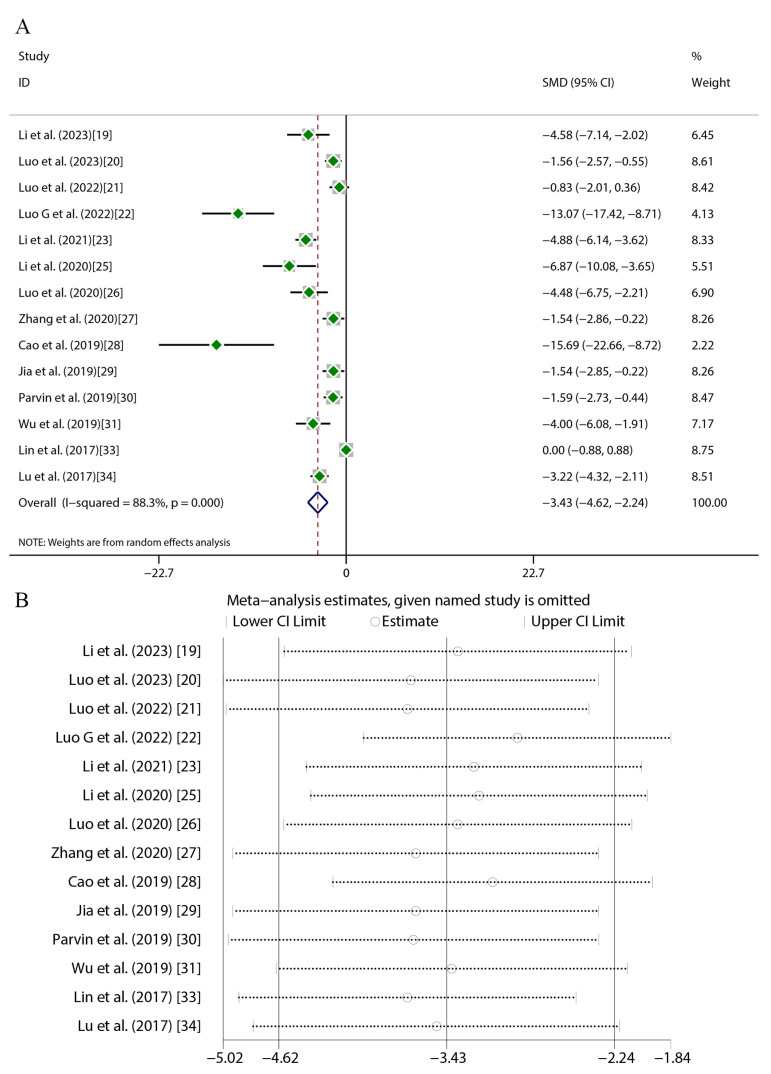
Forest plot (**A**) and sensitivity analysis (**B**) of LDL-C levels [19,20,21,22,23,25,26,27,28,29,30,31,33,34].

Quercetin administration was found to have significant therapeutic effects across key atherosclerosis parameters. For aortic lesion areas (17 studies), random-effects meta-analysis revealed substantial heterogeneity (*I*^2^ = 84.9%, *p* < 0.001), with a marked reduction versus the controls (SMD = −4.16, 95%CI [−5.41, −2.91], *p* < 0.01) (Figure 4A). TC (16 studies) exhibited greater heterogeneity (*I*^2^ = 89.3%, *p* < 0.001) but exhibited a significant decrease (SMD = −2.56, 95%CI [−3.48, −1.64], *p* < 0.01) (Figure 5A). TG levels (18 studies) were similarly reduced (SMD = −1.94, 95%CI [−2.70, −1.19], *p* < 0.01; *I*^2^ = 87%, *p* < 0.001) (Figure 6A). HDL-C (12 studies) showed a significant elevation (SMD = 1.49, 95%CI [0.45, 2.54], *p* < 0.01) despite high heterogeneity (*I*^2^ = 88.6%, *p* < 0.001) (Figure 7A). LDL-C (14 studies) exhibited the strongest reduction (SMD = −3.43, 95%CI [−4.62, −2.24], *p* < 0.01; *I*^2^ = 88.3%, *p* < 0.001) (Figure 8A). Sensitivity analyses confirmed result stability across all parameters, as evidenced by consolidated effect estimates remaining within overall confidence intervals (Figure 4B, Figure 5B, Figure 6B, Figure 7B and Figure 8B).

#### 3.4.2. Secondary Outcomes

##### Inflammation-Related Indicators

Meta-analysis of inflammatory biomarkers revealed quercetin’s significant immunomodulatory effects: IL-1β (five studies; SMD = −4.46, 95%CI [−7.25, −1.67], *p* < 0.01; *I*^2^ = 90.9%), IL-6 (six studies; SMD = −3.98, [−5.57, −2.38], *p* < 0.01; *I*^2^ = 79.3%), and IL-18 (three studies; SMD = −6.70, [−11.64, −1.76], *p* < 0.01; *I*^2^ = 92.4%) exhibited significant reductions under random-effects models due to high heterogeneity (*p* < 0.01). IL-10 exhibited elevations (four studies; SMD = 1.99, [0.21, 3.78], *p* < 0.05; *I*^2^ = 82.4%). Reductions in TNF-α levels were consistent (seven studies; SMD = −4.34, [−5.84, −2.83], *p* < 0.01; *I*^2^ = 71.4%). Levels of the adhesion molecules VCAM-1 (five studies; SMD = −2.47, [−4.33, −0.60], *p* < 0.05; *I*^2^ = 78.3%) and ICAM-1 (two studies; SMD = −3.30, [−4.79, −1.82], *p* < 0.01; fixed-effects; *I*^2^ = 30.8%) decreased significantly, while MCP-1 levels declined markedly (two studies; SMD = −4.48, [−6.30, −2.66], *p* < 0.01; fixed-effects; *I*^2^ = 15.2%). F4/80 showed a non-significant reduction trend (two studies; SMD = −9.46, [−25.79, 6.87], *p* > 0.05; random-effects; *I*^2^ = 83.3%). Collagen fibre area exhibited a non-significant increase (five studies; SMD = 2.87, [−0.18, 5.91], *p* > 0.05; *I*^2^ = 93.9%) (Table 3).

##### Oxidative-Stress-Related Indicators

Quercetin significantly modulated key oxidative stress markers: malondialdehyde (MDA) levels decreased across four heterogeneous studies (*I*^2^ = 89.1%; random-effects SMD = −2.48, 95%CI [−4.30, −0.66], *p* < 0.01), whereas superoxide dismutase (SOD) activity increased in three homogeneous studies (*I*^2^ = 0%; fixed-effects SMD = 1.79, [1.13, 2.45], *p* < 0.01). Catalase (CAT) elevation reached statistical significance in two concordant reports (*I*^2^ = 9.3%; fixed-effects SMD = 0.95, [0.05, 1.86], *p* < 0.05). Although glutathione peroxidase (GPX) showed an increasing trend in three highly heterogeneous studies (*I*^2^ = 94.4%; random-effects SMD = 4.29, [−0.31, 8.89]), this did not achieve statistical significance (*p* > 0.05) (Table 3).

##### Effects on Lipid Metabolism

Quercetin significantly modulated lipid metabolism markers: a lipid area reduction was observed (four studies; random-effects SMD = −5.40, 95%CI [−10.29, −0.51], *p* < 0.05; *I*^2^ = 91.2%), while ATP-binding cassette transporters exhibited differential regulation—ABCA1 levels increased substantially (four studies; SMD = 7.51, [2.98, 12.05], *p* < 0.01; *I*^2^ = 80.0%), whereas ABCG1 levels showed a non-significant elevation (two studies; SMD = 2.49, [−1.49, 6.46], *p* > 0.05; *I*^2^ = 75.0%). Oxidized LDL levels decreased significantly (three studies; SMD = −9.47, [−17.72, −1.23], *p* < 0.05; *I*^2^ = 85.5%). CD36 reduction was consistent across homogeneous studies (two studies; fixed-effects SMD = −3.72, [−5.29, −2.16], *p* < 0.01; *I*^2^ = 0%) (Table 3).

##### Mechanisms Protecting Against Atherosclerosis

Quercetin significantly modulated key molecular targets: Liver X Receptor α (LXRα) levels exhibited elevation (four studies; fixed-effects SMD = 2.77, 95%CI [1.79, 3.76], *p* < 0.01; *I*^2^ = 22.5%), while haem oxygenase-1 (HO-1) levels increased under high heterogeneity (three studies; random-effects SMD = 4.20, [0.17, 8.23], *p* < 0.05; *I*^2^ = 93.9%). Sirtuin 1 (SIRT1) showed significant upregulation (three studies; SMD = 3.24, [2.03, 4.46], *p* < 0.01; fixed-effects). Conversely, mechanistic target of rapamycin (mTOR) levels decreased (two studies; SMD = −2.74, [−4.34, −1.15], *p* < 0.01; fixed-effects; *I*^2^ = 0%), as did levels of proprotein convertase subtilisin/kexin type 9 (PCSK9) (SMD = −5.27, [−7.15, −3.39], *p* < 0.01; fixed-effects; *I*^2^ = 62.1%). NADPH oxidase components exhibited consistent suppression—NADPH (SMD = −6.97, [−9.28, −4.65], *p* < 0.01), NOX2 (SMD = −1.50, [−2.43, −0.57], *p* < 0.01), and p47phox (three studies; SMD = −5.54, [−7.29, −3.79], *p* < 0.01)—all under fixed-effects models (*I*^2^ = 0%). Reductions in P21 reached statistical significance despite heterogeneity (two studies; random-effects SMD = −12.68, [−24.36, −1.01], *p* < 0.05; *I*^2^ = 84.4%) (Table 3).

### 3.5. Heterogeneity and Results of Subgroup Analysis

Potential sources of heterogeneity, including divergent modelling approaches, animal species variability, and differential treatment durations, were investigated through stratified subgroup analyses of aortic lesion areas, TC, TG, and LDL-C across seven dimensions (language type, species, gender, modelling duration, administration period, dosage, and method). For aortic lesions, heterogeneity was substantially lower in Chinese-language publications (vs. those in English), ≤8-week and ≥16-week modelling cohorts, and corresponding administration periods; intermediate durations (8–16 weeks) showed no significant heterogeneity reduction. TC heterogeneity demonstrated significant attenuation in Wistar rats, ≤8-week modelling cohorts, ≤8-week treatment groups, orally administered interventions, and high-dose regimens (≥500 mg/kg). TG heterogeneity was notably lower in Chinese publications, Wistar rats, and mid-duration modelling cohorts (8–16 weeks). LDL-C heterogeneity was primarily attributable to species variation, with significant reductions exclusively observed in Wistar rat subgroups (Table 4). Despite conducting stratified subgroup analyses of aortic lesion areas, TC, TG, and LDL-C across seven dimensions to explore potential sources of heterogeneity, substantial heterogeneity persisted in several subgroups. Accordingly, the pooled effect-size estimates derived from this meta-analysis should be interpreted with strict caution, and the present findings are intended to generate testable hypotheses rather than confirm definitive conclusions regarding the protective effects of quercetin in atherosclerotic animal models.

### 3.6. Publication Bias

**Figure 9:**
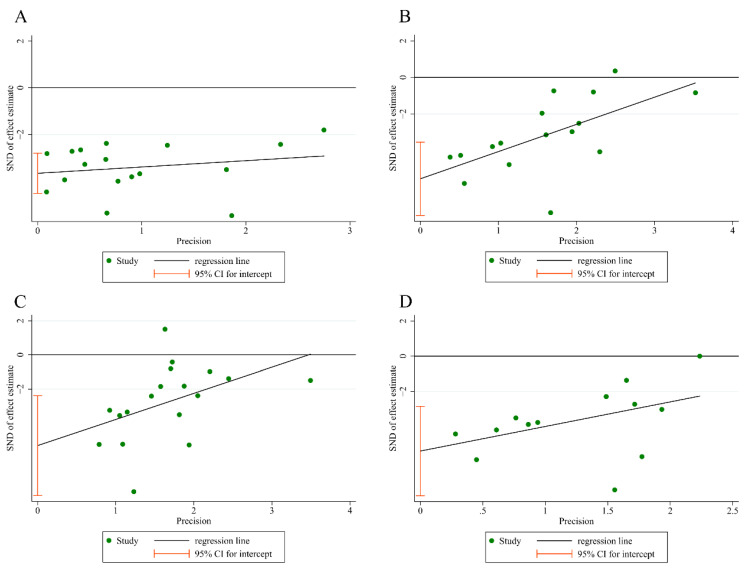
Egger’s publication bias plot for (**A**) aortic lesion areas; (**B**) TC; (**C**) TG; and (**D**) LDL-C.

We conducted Egger’s analysis on four main indicators: aortic lesion areas, TC, TG, and LDL-C. The results of the Egger’s analysis indicated that there was a certain degree of publication bias in the selected studies (aortic lesion areas: *p* > |t| = 0.000 < 0.05; TC: *p* > |t| = 0.000 < 0.05; TG: *p* > |t| = 0.001 < 0.05; LDL-C: *p* > |t| = 0.001 < 0.05) (Figure 9).

### 3.7. Time–Dose Interval Analysis

**Figure 10:**
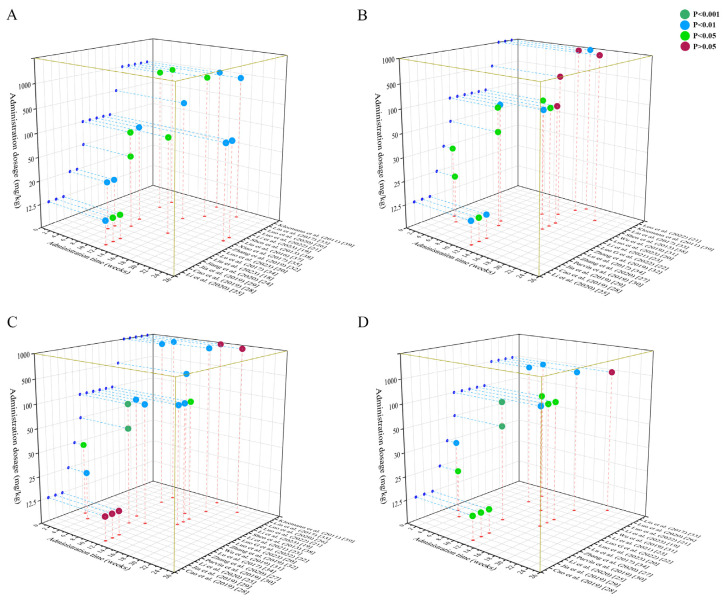
Time–dose interval analysis scatter plot for (**A**) Aortic lesion areas; (**B**) TC; (**C**) TG; and (**D**) LDL-C. This figure displays three-dimensional scatter plots of time–dose interval analysis, illustrating the association between quercetin administration (dosage and weeks) and the statistical significance of its effects on key outcomes in atherosclerotic animal models. Blue—*p* < 0.01 (statistically significant effect, strong evidence); green—*p* < 0.05 (statistically significant effect, moderate evidence); red—*p* > 0.05 (no statistically significant effect) [18,19,20,21,22,23,24,25,26,27,28,29,30,31,32,33,34,35,37,38,39].

Dose–time response surface modelling (Figure 10) elucidated quercetin’s therapeutic window for core atherosclerosis parameters. Aortic lesion improvement required doses of 12.5–1000 mg/kg over 8–24 weeks. TC reduction exhibited a narrower effective range (12.5–100 mg/kg; 2–12 weeks), with diminished efficacy beyond 100 mg/kg or after 12 weeks. TG modulation occurred at 25–1000 mg/kg over 2–16 weeks, though efficacy lapsed at 12/15-week intervals. LDL-C reduction spanned 12.5–1000 mg/kg across 2–16 weeks. Optimal intervention parameters converging across all endpoints were established at 25–100 mg/kg administered for 8–10 weeks, achieving maximal therapeutic consistency for atherosclerotic pathology mitigation.

### 3.8. Trial Sequential Analysis

**Figure 11:**
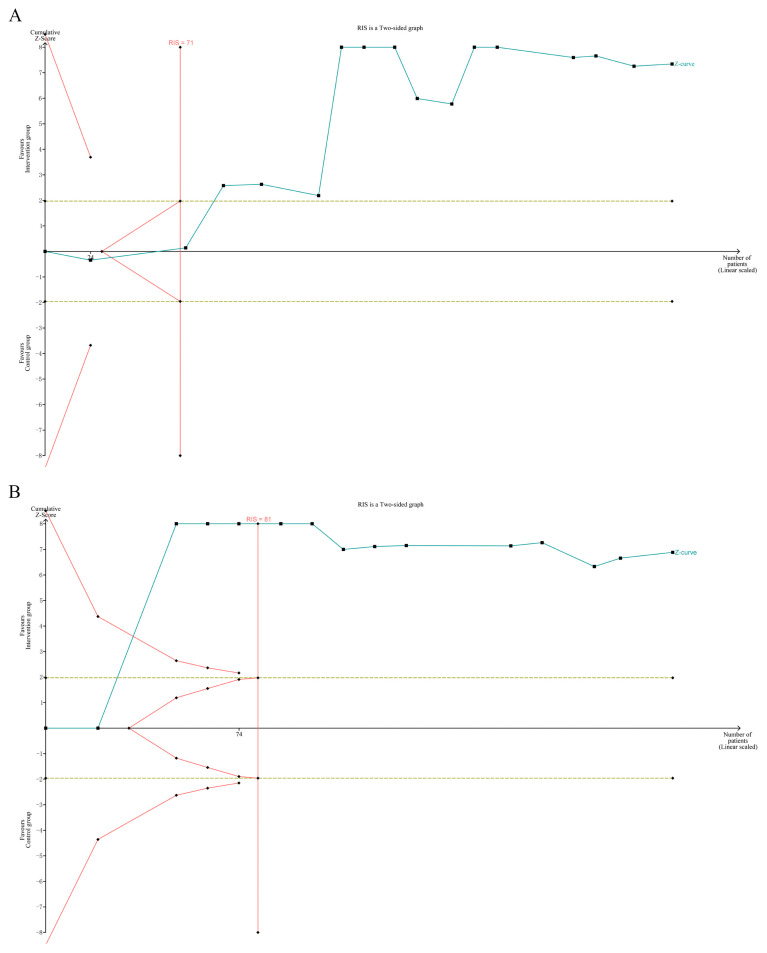
TSA for the effects of quercetin on reducing (**A**) TC; (**B**) LDL-C.

To eliminate false-positive results, we conducted a trial sequential analysis on the two main outcome indicators, TC and LDL-C. As shown in Figure 11A, the trial sequential analysis-adjusted significance boundary (RIS) for this graph was 71, with the cumulative Z-curve crossing both the traditional boundary (Z = 1.96) and the RIS boundary, reaching the expected information size. In Figure 11B, the RIS for this graph was 81, with the cumulative Z-curve also crossing the traditional boundary (Z = 1.96) and the RIS boundary, achieving the expected information size. Therefore, the quercetin intervention group exhibited superior performance relative to the control group in terms of TC and LDL-C, and the trial sequential analysis confirmed the statistical sufficiency of these findings within the context of current preclinical animal studies. However, given the presence of significant publication bias (identified via Egger’s test), substantial residual heterogeneity, and the preclinical nature of the included data, this conclusion should be interpreted with caution. The TSA results verify the statistical robustness of the current dataset rather than establishing definitive clinical certainty, and further validation through well-designed studies will help consolidate these observations.

## 4. Discussion

### 4.1. Effectiveness and Summary of Evidence

**Figure 12:**
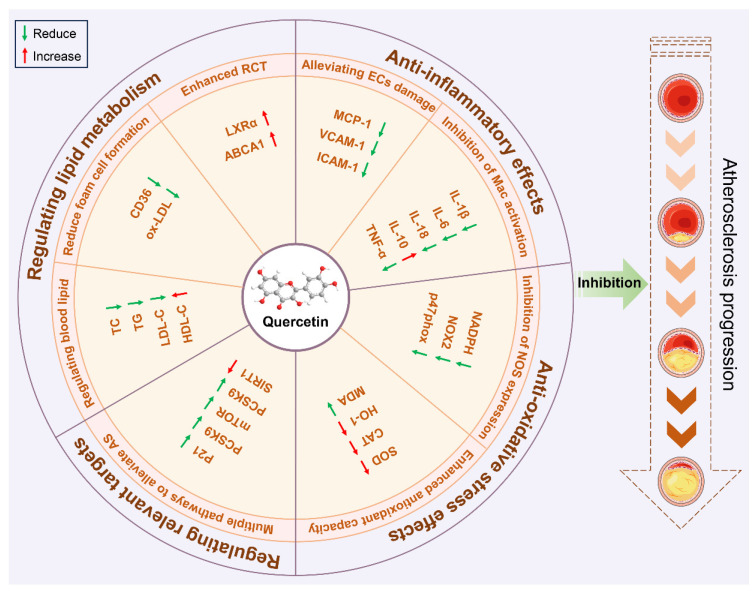
Quercetin regulates related outcome indicators.

The primary objective of this study was to determine the therapeutic effects of quercetin on atherosclerosis and elucidate its molecular biological mechanisms. A total of 22 studies involving 421 animals were included in this review, with retrieved research quality generally rated as moderate or above average. A meta-analysis of data from 22 preclinical studies indicated that quercetin improved relevant outcome measures. Its specific mechanisms may involve regulating lipid metabolism, exerting anti-inflammatory and antioxidant effects, and modulating key targets (Figure 12).

### 4.2. Potential Mechanism of Quercetin in Treating Atherosclerosis

**Figure 13:**
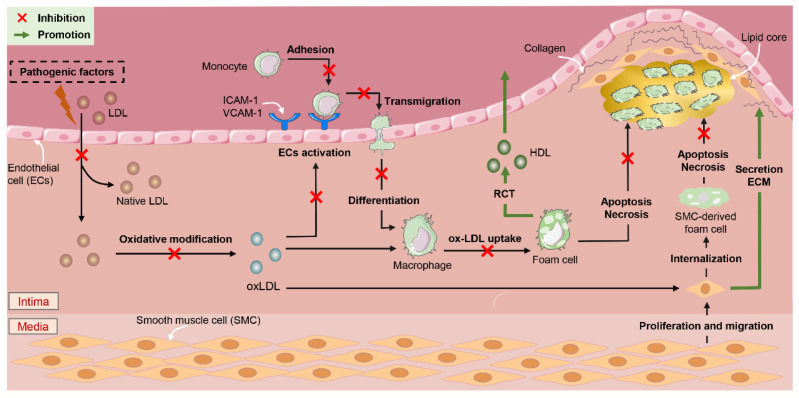
Potential mechanisms whereby quercetin ameliorates AS in animals.

Endothelial cells (ECs), macrophages (Macs), and vascular smooth muscle cells (VSMCs) are the main cell populations involved in the pathogenesis of AS [40]. The intact ECs layer maintains a complex functional balance to inhibit inflammatory responses or thrombosis [41]. An inflammatory response induced by macrophages and the formation of macrophage-derived foam cells are the main events in the occurrence and development of atherosclerosis [42]. VSMCs are key participants in advanced atherosclerosis: on the one hand, the migration of VSMCs from the arterial medium to arterial intima leads to an increase in the levels of VSMC-derived foam cells [43]; on the other hand, the protective fibrous cap formed by its secreted extracellular matrix (ECM) covers the “necrotic” core and is considered to be a plaque stabilizer [44]. Our meta-analysis and systematic evaluation results showed that quercetin played a protective role mainly by interfering with the three cell populations above (Figure 13).

#### 4.2.1. Regulating Lipid Metabolism

Endothelial injury leads to accumulation and infiltration of LDL-C particles under the endothelium, an important early predictor of atherosclerosis [45]. Hyperlipidaemia is one of the main factors leading to endothelial injury [46]. Hyperlipidaemia is characterized by a decrease in HDL-C levels and an increase in TC, TG, and LDL-C levels in the blood, in which LDL-C plays a major role in the formation of atherosclerotic plaques [47]. This meta-analysis demonstrates that quercetin significantly modulates serum lipid profiles, evidenced by reductions in TC, TG, and LDL-C concomitant with elevated HDL-C levels.

The progress of atherosclerosis is related to the uptake of lipoproteins by macrophages and their transformation into foam cells [48]. Macrophages upregulate pattern recognition receptors, including scavenger receptors (such as CD36), and phagocytize a large number of oxLDLs, leading to intracellular lipid accumulation and the formation of foam cells. Foam cells accumulate to form lipid stripes and even more complex atherosclerotic plaques [49,50]. Our meta-analysis indicates that quercetin reduces foam cell formation by decreasing CD36 expression and ox-LDL production.

There is a cholesterol-scavenging mechanism in foam cells, which is called reverse cholesterol transport (RCT). Liver X receptor (LXR) is a key sterol-sensitive transcription factor regulating intracellular cholesterol in macrophages that is activated by excessive cholesterol levels. LXR in turn drives and mediates the expression of genes in the cholesterol efflux pathway, including ABCA1 and ABCG1, which are key receptors in the initial step of RCT [51,52]. Peroxisome proliferator-activated receptor gamma (PPARγ) is a key regulator of lipid metabolism. Circulating oxLDL strongly activates PPARγ, thereby activating the PPARγ-LXRα-ABC transporter pathway and enhancing cholesterol efflux [53,54]. Many studies have confirmed that quercetin can improve fat accumulation by targeting the PPARγ—LXRα—ABC pathway [55,56]. The low-density lipoprotein receptor (LDLR) serves to uptake and internalize lipoproteins, particularly LDL-C [57]. Studies have shown that PCSK9 promotes LDLR degradation and increases circulating LDL-C levels by binding to LDLR [58]. An experiment conducted on RAW264.7 macrophages showed that quercetin inhibited oxLDL-induced lipid droplets in RAW264.7 cells by upregulating the levels of ABCA1, ABCG1, and LXRα and downregulating the expression of PCSK9 [56]. Our meta-analysis results indicate that quercetin enhances anti-atherosclerotic effects by increasing LXRα and ABCA1 expression levels, decreasing PCSK9 expression levels, and reducing aortic lipid and plaque area. This mechanism enhances the RCT pathway and lowers circulating LDL-C levels.

#### 4.2.2. Anti-Inflammatory Effects

Under normal physiological conditions, ECs are closely arranged in the vascular intima, which can inhibit inflammatory responses and reduce lipid deposition and thrombosis [41,59]. Various pathogenic factors can cause vascular EC activation, dysfunction, and loss of integrity, resulting in excessive numbers of components in the blood entering the arterial wall, and the low-density lipoprotein (LDL) deposited in the inner layer of the vascular wall is oxidized to oxidized low-density lipoprotein (oxLDL) [60,61]. Activated endothelial cells release intercellular adhesion molecules (e.g., ICAM-1), vascular intercellular adhesion molecules (e.g., VCAM-1), and chemokines (e.g., MCP-1) and promote the migration of monocytes to the intima and their subsequent transformation into macrophages [62]. Our meta-analysis showed that quercetin significantly reduced the expression levels of ICAM, VCAM-1, and MCP-1. The results confirmed that quercetin could reduce the number of monocytes transformed into Macs in the intima by reducing the adhesion and migration of monocytes in the blood.

Macs play an important role in the phagocytosis of oxLDL, while pro-inflammatory M1 Macs and anti-inflammatory M2 Macs aggravate or alleviate atherosclerosis, respectively [63]. In AS lesions, cholesterol crystals, pro-inflammatory cytokines, and oxLDL can induce pro-inflammatory M1 Mac activity. M1 Macs activate and secrete proinflammatory factors such as tumour necrosis factor-α (TNF-α), IL-1β, IL-6, and IL-18 through the toll-like receptor (TLR-4) or nuclear factor—κB (NF-κB) pathways. On the other hand, IL-4 or IL-13 can activate the polarization of M2 Macs through the JAK-STAT, PPARγ, adenosine monophosphate-activated protein kinase (AMPK), and TGF-β pathways, and M2 Macs produce anti-inflammatory cytokines, such as IL-10 and TGF-β [64,65]. There is evidence that the polarization of M0 MAC (a static Mac is called M0 Mac) to the M1 phenotype can be achieved through the PI3K-AKT-mTOR-HIF1 α (hypoxia inducible factor 1-α) signalling pathway [64]. Several in vitro and in vivo studies highlight the protective effect of SIRT1 on vascular aging. SIRT1 expression is significantly reduced in aging vascular tissues [66,67]. Some studies have shown that the expression of pro-inflammatory cytokines is inhibited by SIRT1, because it can mediate the initiation and progression of inflammation [68]. These findings suggest that quercetin may suppress inflammatory responses by regulating macrophage polarization, increasing SIRT1 expression levels, and reducing mTOR expression levels.

#### 4.2.3. Anti-Oxidative-Stress Effects

The deposition of LDL on the endothelium of the vasculature induces inflammatory responses that generate large quantities of reactive oxygen species (ROS). LDL particles can be oxidized by ROS into oxLDL, and oxLDL stimulates subsequent inflammatory responses [69]. In response to stress caused by sensitive factors such as ROS, oxLDL, and lipid peroxidation, a nuclear factor E2-related factor 2 (NRF2)-mediated transcription process is initiated to activate a series of antioxidant enzymes, such as HO-1, SOD, and GPX, to scavenge reactive oxygen species and other harmful substances and promote anti-oxidative stress, anti-inflammatory, anti-apoptotic, and other cellular-protective mechanisms [70,71]. Multiple studies have demonstrated that quercetin alleviates the damage caused by a variety of factors by modulating the NRF2/HO-1 pathway in response to oxidative stress injuries [72,73,74]. Our meta-analysis results showed that quercetin significantly decreased the levels of oxLDL, NADPH, NOX2, p47phox, and MDA and significantly increased the levels of antioxidant enzymes such as HO-1, CAT, and SOD. The results above suggest that quercetin inhibits oxidative stress in AS by decreasing oxidative modification of LDL, reducing ROS production, and modulating the NRF2/HO-1 pathway.

### 4.3. Challenges in Translating Preclinical Quercetin Doses into Human Applications

It is noteworthy that the dosage range of quercetin employed in preclinical studies was considerably broad (12.5–1000 mg/kg), and this wide variation in dosage may reduce the credibility of pooled results. Such dose heterogeneity primarily relates to differences in animal strains, modelling protocols, and intervention duration. Consequently, we analysed the dose–response relationship of quercetin across all included studies. The results indicate that the most pronounced improvement in primary outcome measures occurs within the 25–100 mg/kg dosage range. However, the optimal dosage established in this animal study also presents one of the key obstacles to clinical translation—namely, a significant mismatch between this dosage and the feasible dietary quercetin intake achievable in humans. When we converted doses based on a formula recommended in the literature [75], this preclinical range substantially exceeds quercetin levels achievable from dietary sources, limiting supplementation through food sources. Secondly, in clinical applications, quercetin exhibits relatively poor bioavailability due to factors such as its low water solubility and short metabolic half-life [76]. These factors are frequently circumvented in animal studies through specialised formulations not yet widely used clinically. Consequently, future research should focus on investigating cross-species dose-scaling strategies and high-bioavailability formulations to address these gaps.

### 4.4. Limitations

Several methodological limitations warrant consideration prior to clinical translation of these findings. First, our exclusive reliance on Chinese and English databases potentially introduced language bias in source selection. Second, the indirect data extraction methods employed for partial dataset acquisition may have led to quantification discrepancies. Third, the CAMARADES assessment identified key methodological flaws in the included studies: inadequate assessment of blinded outcomes, poor description of allocation concealment, and undisclosed anaesthetic techniques in some cases; these methodological shortcomings may have inflated estimates of the combined effect size. Fourth, our exclusive inclusion of the highest quercetin dose in multi-dose studies may have led to an overestimation of therapeutic efficacy and impeded comprehensive interpretation of the dose–response relationship, representing a key limitation of this meta-analysis. Fifth, despite subgroup analyses addressing heterogeneity sources, substantial residual heterogeneity persists in terms of aortic lesion area, TC, TG, and LDL-C metrics. Finally, while TSA confirmed the results’ stability, Egger’s test detected publication bias among core outcome measures.

## 5. Conclusions

In summary, our results suggest that quercetin improves plaque area and lipid levels in atherosclerotic animals, showing optimal efficacy when administered at a dose of 25–100 mg/kg and a dry period of 8–10 weeks. Quercetin exerts protective effects at multiple points in the development of atherosclerosis, including regulating blood lipid levels to reduce endothelial damage, inhibiting the release of adhesion factors to reduce the number of monocytes converted to macrophages, modulating the NADPH/HO-1 pathway to enhance the body’s antioxidant capacity to reduce the production of oxLDL, modulating the mTOR/SIRT1 pathway to reduce the release of pro-inflammatory factors in order to inhibit the immune responses of macrophages, and modulating the LXRα-ABC metabolic pathway to enhance RCT in order to reduce foam cell formation. Based on the evidence above, quercetin may serve as a potential and promising natural drug against atherosclerosis. However, basic research on animal models does not represent clinical practice; it only serves as a useful supplement to preclinical evidence.

## Figures and Tables

**Table 1:** Characteristics of the included studies.

Studies	Language Type	Species (Sex, *n* = I/C Group, Weight)	Control Group (Method)	Intervention Group (Method)	Anaesthetic	Outcomes	Intergroup Difference
Liu et al. (2023) [18]	English	ApoE^−/−^ mice (male, 7/7, 18–22 g)	HFD for 12 weeks	Qu (20 mg/kg) intragastric administration for 8 weeks	Pentobarbital sodium	Aortic lesion areas Sirt1 P21 F4/80	*p* < 0.05 *p* < 0.05 *p* < 0.05 *p* < 0.05
							
Li et al. (2023) [19]	English	ApoE^−/−^ mice (male, 6/6)	HFD for 8 weeks	Qu (1000 mg/kg) oral administration for 8 weeks	NM	Aortic lesion areas TG LDL-C IL-1β VCAM-1 NADPH Nox2 p47phox	*p* < 0.01 *p* < 0.05 *p* < 0.05 *p* < 0.05 *p* < 0.05 *p* < 0.01 *p* > 0.05 *p* < 0.01
Luo et al. (2023) [20]	English	ApoE^−/−^ mice (female, 10/10, 18–20 g)	HFD for 16 weeks	Qu (100 mg/kg) intragastric administration for 16 weeks	NM	Aortic lesion areas TC TG LDL-C HDL-C TNF-α VCAM-1 MCP-1	*p* < 0.01 *p* > 0.05 *p* < 0.05 *p* < 0.05 *p* < 0.05 *p* < 0.01 *p* < 0.01 *p* < 0.01
							
Luo X et al. (2022) [21]	English	ApoE^−/−^ mice (male, 6/6, 6–8 weeks)	HFD for 16 weeks	Qu (1000 mg/kg) oral administration for 16 weeks	Pentobarbital sodium	Aortic lesion areas Lipid areas TC TG LDL-C HDL-C IL-18 IL-1β	*p* < 0.01 *p* < 0.05 *p* < 0.05 *p* < 0.05 *p* < 0.05 *p* < 0.05 *p* < 0.01 *p* < 0.01
							
Luo G et al. (2022) [22]	Chinese	ApoE^−/−^ mice (female, 10/10)	HFD for 16 weeks	Qu (100 mg/kg) intragastric administration for 16 weeks	Pentobarbital sodium	TC TG LDL-C HDL-C ABCA1 ABCG1 CD36 LXRα	*p* < 0.05 *p* < 0.05 *p* < 0.01 *p* < 0.05 *p* < 0.05 *p* > 0.05 *p* < 0.01 *p* < 0.05
							
Li et al. (2021) [23]	English	ApoE^−/−^ mice (male, 20/20, 6 weeks)	HFD for 16 weeks	Qu (100 mg/kg) intragastric administration for 16 weeks	Pentobarbital sodium	TC TG HDL-C LDL-C	*p* < 0.01 *p* < 0.01 *p* < 0.01 *p* < 0.01
Jiang et al. (2020) [24]	English	ApoE^−/−^ mice (male, 7/7, 6 weeks)	HFD for 12 weeks	Qu (20 mg/kg) intragastric administration for 8 weeks	Pentobarbital sodium	Aortic lesion areas IL-6 VCAM-1 ICAM-1 Sirt1	*p* < 0.05 *p* < 0.05 *p* < 0.05 *p* < 0.05 *p* < 0.05
							
Li et al. (2020) [25]	English	ApoE^−/−^ mice (male, 6/6, 12 weeks)	HFD for 12 weeks	Qu (12.5 mg/kg) intragastric administration for 12 weeks	Pentobarbital sodium	Aortic lesion areas Lipid areas TC TG LDL-C HDL-C TNF-α IL-6 IL-10 ABCA1 LXRα PCSK9	*p* < 0.05 *p* < 0.01 *p* < 0.05 *p* > 0.05 *p* < 0.01 *p* > 0.05 *p* < 0.01 *p* < 0.01 *p* < 0.01 *p* < 0.01 *p* < 0.01 *p* < 0.01
							
Luo et al. (2020) [26]	English	ApoE^−/−^ mice (male, 6/6, 6 weeks)	HFD for 8 weeks	Qu (1000 mg/kg) oral administration for 8 weeks	NM	Aortic lesion areas TG LDL-C NADPH HO-1 Nox2 p47phox	*p* < 0.01 *p* < 0.05 *p* < 0.05 *p* < 0.01 *p* < 0.01 *p* > 0.05 *p* < 0.01
							
Zhang et al. (2020) [27]	English	Wistar rat (male, 6/6, 230–270 g)	HFD for 8 weeks	Qu (30 mg/kg) intragastric administration for 2 weeks	Pentobarbital sodium	TC TG LDL-C HDL-C IL-1β IL-10 MDA SOD CAT GPX Sirt1	*p* < 0.01 *p* < 0.01 *p* < 0.05 *p* > 0.05 *p* < 0.05 *p* < 0.05 *p* < 0.01 *p* < 0.01 *p* < 0.05 *p* < 0.05 *p* < 0.01
							
Cao et al. (2019) [28]	English	ApoE^−/−^ mice (male, 6/6, 12 weeks)	HFD for 12 weeks	Qu (12.5 mg/kg) intragastric administration for 12 weeks	Pentobarbital sodium	Aortic lesion areas Lipid areas TC TG LDL-C HDL-C TNF-α IL-1β IL-18 mTOR P21	*p* < 0.01 *p* < 0.01 *p* < 0.01 *p* > 0.05 *p* < 0.01 *p* > 0.05 *p* < 0.01 *p* < 0.01 *p* < 0.01 *p* < 0.05 *p* < 0.05
							
Jia et al. (2019) [29]	English	ApoE^−/−^ mice (male, 6/6, 12 weeks)	HFD for 12 weeks	Qu (12.5 mg/kg) intragastric administration for 12 weeks	Pentobarbital sodium	Aortic lesion areas Lipid areas TC TG LDL-C HDL-C TNF-α IL-6 IL-10 CD36 LXRα ABCA1 PCSK9 oxLDL	*p* < 0.01 *p* < 0.05 *p* < 0.05 *p* > 0.05 *p* < 0.01 *p* > 0.05 *p* < 0.01 *p* < 0.01 *p* < 0.01 *p* < 0.05 *p* < 0.05 *p* < 0.01 *p* < 0.05 *p* < 0.001
							
Parvin et al. (2019) [30]	English	Wistar rat (male, 8/8, ~180 g)	HFD for 40 days	Qu (25 mg/kg) intragastric administration for 4 weeks	Ketamine– Xylazine	TC TG LDL-C HDL-C MDA	*p* < 0.01 *p* < 0.05 *p* < 0.01 *p* < 0.05 *p* < 0.01
							
Wu et al. (2019) [31]	English	ApoE^−/−^ mice (male, 6/6, 4–8 weeks)	HFD for 12 weeks	Qu (100 mg/kg) intragastric administration for 12 weeks	NM	TC TG LDL-C HDL-C TNF-α IL-6	*p* < 0.01 *p* < 0.05 *p* < 0.01 *p* < 0.01 *p* < 0.01 *p* < 0.01
							
Zhang et al. (2019) [32]	Chinese	ApoE^−/−^ mice (female, 9/9, 18.8–20.2 g)	HFD for 8 weeks	Qu (100 mg/kg) intragastric administration for 9 weeks	Isoflurane	Aortic lesion areas TC TG MCP-1	*p* < 0.05 *p* < 0.05 *p* < 0.05 *p* < 0.05
							
Lin et al. (2017) [33]	English	ApoE^−/−^ mice (male, 10/10, 6–8 weeks)	AIN-93G diets for 20 weeks	Qu (1000 mg/kg) oral administration for 20 weeks	NM	Aortic lesion areas TC TG LDL-C HDL-C IL-6 IL-10 F4/80	*p* < 0.05 *p* > 0.05 *p* > 0.05 *p* > 0.05 *p* > 0.05 *p* < 0.01 *p* < 0.05 *p* < 0.01
							
Lu et al. (2017) [34]	English	C57BL/6 mice (male, 15/15, 18–22 g)	High-fructose diets for 16 weeks	Qu (50/100 mg/kg) intragastric administration for 10 weeks	NM	Aortic lesion areas TC TG LDL-C HDL-C MDA SOD TNF-α IL-1β IL-18 IL-6 HO-1	*p* < 0.01 *p* < 0.01 *p* < 0.001 *p* < 0.001 *p* < 0.001 *p* < 0.01 *p* < 0.01 *p* < 0.001 *p* < 0.001 *p* < 0.001 *p* < 0.001 *p* < 0.001
							
Xiao et al. (2017) [35]	English	ApoE^−/−^ mice (male, 15/15, 18–20 g)	HFD for 24 weeks	Qu (25/50/100 mg/kg) oral administration for 24 weeks	NM	Aortic lesion areas MDA GPX oxLDL p47phox	*p* < 0.05 *p* > 0.05 *p* < 0.01 *p* < 0.01 *p* < 0.01
							
Guo et al. (2016) [36]	English	ApoE^−/−^ mice (male, 10/10, 18–22 g)	HFD	Qu (12.5 mg/kg) intragastric administration	NM	Aortic lesion areas TC TG ABCA1 ABCG1 LXRα SOD TNF-α GPX CAT VCAM-1 ICAM-1 oxLDL	*p* < 0.01 *p* < 0.05 *p* < 0.05 *p* < 0.05 *p* < 0.05 *p* < 0.05 *p* < 0.05 *p* < 0.05 *p* < 0.05 *p* < 0.05 *p* > 0.05 *p* < 0.01 *p* < 0.01
							
Liu et al. (2016) [37]	Chinese	ApoE^−/−^ mice (male, 4/4, 6 weeks)	HFD for 24 weeks	Qu (25/50/100 mg/kg) intragastric administration for 24 weeks	Pentobarbital sodium	Aortic lesion areas mTOR	*p* < 0.05 *p* < 0.05
							
Shen et al. (2013) [38]	English	ApoE^−/−^ mice (male, 25/25, 4 weeks)	HFD for 14 weeks	Qu (500 mg/kg) oral administration for 14 weeks	Isoflurane	Aortic lesion areas TC TG HO-1	*p* < 0.05 *p* > 0.05 *p* < 0.05 *p* < 0.05
							
Kleemann et al. (2011) [39]	English	ApoE*3-Leiden mice (female, 12/13)	HFD for 15 weeks	Qu (1000 mg/kg) oral administration for 15 weeks	NM	Aortic lesion areas TC TG VCAM-1	*p* < 0.05 *p* > 0.05 *p* > 0.05 *p* > 0.05

**Table 2:** Quercetin information for each study.

Studies	Source	Purity (%)	Quality Control Reported
Liu et al. (2023) [18]	Dalian Meilun Biotechnology Co., Ltd., Dalian, China	Unknown	HPLC
Li et al. (2023) [19]	Sigma-Aldrich, USA	≥95	HPLC
Luo et al. (2023) [20]	Sigma-Aldrich, St Louis, MO, USA	98	HPLC
Luo X et al. (2022) [21]	Chengdu Herbpurify, Chengdu, China	98	HPLC
Luo G et al. (2022) [22]	Sigma, USA	Unknown	HPLC
Li et al. (2021) [23]	Unknown	Unknown	Unknown
Jiang et al. (2020) [24]	Jiashi Yuhe Institute, Beijing, China	98.6	HPLC
Li et al. (2020) [25]	Shanghai Yuanye Biotechnology Co., Ltd., Shanghai, China	>98	HPLC
Luo et al. (2020) [26]	Sigma-Aldrich, USA	≥95	HPLC
Zhang et al. (2020) [27]	Sigma-Aldrich, USA	Unknown	HPLC
Cao et al. (2019) [28]	Shanghai Yuanye Biotechnology Co., Ltd., Shanghai, China	≥98	HPLC
Jia et al. (2019) [29]	Shanghai Yuanye Biotechnology Co., Ltd., Shanghai, China	≥98	HPLC
Parvin et al. (2019) [30]	Unknown	Unknown	Unknown
Wu et al. (2019) [31]	Unknown	Unknown	Unknown
Zhang et al. (2019) [32]	Sinopharm Chemical Reagent Co., Ltd., China	98.8	HPLC
Lin et al. (2017) [33]	Unknown	Unknown	Unknown
Lu et al. (2017) [34]	Sigma-Aldrich, USA	>98	HPLC
Xiao et al. (2017) [35]	Sigma-Aldrich, St Louis, Missouri, USA	98	HPLC
Guo et al. (2016) [36]	Unknown	Unknown	Unknown
Liu et al. (2016) [37]	Sigma-Aldrich, USA	≥95	HPLC
Shen et al. (2013) [38]	Sigma Life Science	>95	HPLC
Kleemann et al. (2011) [39]	Sigma-Aldrich, Steinheim, Germany	Unknown	HPLC

**Table 3:** Comparison of secondary outcome measures of quercetin treatment for atherosclerosis.

Outcome Indicators	Studies (*n*)	T/M (*n*)	Heterogeneity		SMD (95% CI)	*p*-Value
			*I*^2^ (%)	*p*		
(1) Inflammation-related indicators						
IL-1β	5	38/38	90.9	<0.001	−4.46 (−7.25, −1.67)	<0.01
IL-6	6	50/50	79.3	<0.001	−3.98 (−5.57, −2.38)	<0.01
IL-10	4	28/28	81.6	<0.01	1.99 (0.21, 3.78)	<0.05
IL-18	3	27/27	92.4	<0.001	−6.70 (−11.64, −1.76)	<0.01
TNF-α	7	48/48	71.4	<0.01	−4.34 (−5.84, −2.83)	<0.01
VCAM-1	5	27/28	78.3	<0.01	−2.47 (−4.33, −0.60)	<0.05
ICAM-1	2	10/10	30.8	>0.05	−3.30 (−4.79, −1.82)	<0.01
MCP-1	2	10/10	15.2	>0.05	−4.48 (−6.30, −2.66)	<0.01
F4/80	2	13/13	83.3	<0.05	−9.46 (−25.79, 6.87)	>0.05
Collagen fibre area	5	38/38	93.9	<0.001	2.87 (−0.18, 5.91)	>0.05
(2) Oxidative-stress-related indicators						
MDA	4	44/44	89.1	<0.001	−2.48 (−4.30, −0.66)	<0.01
SOD	3	26/26	0.0	>0.05	1.79 (1.13, 2.45)	<0.01
CAT	2	11/11	9.3	>0.05	0.95 (0.05, 1.86)	<0.05
GPX	3	26/26	94.4	<0.001	4.29 (−0.31, 8.89)	<0.05
(3) Effects on lipid metabolism						
Lipid areas	4	23/23	91.2	<0.001	−5.40 (−10.29, −0.51)	<0.05
ABCA1	4	19/19	80.0	<0.01	7.51 (2.98, 12.05)	<0.01
ABCG1	2	7/7	75.0	<0.05	2.49 (−1.49, 6.46)	>0.05
ox-LDL	3	14/14	85.5	<0.05	−9.47 (−17.72, −1.23)	<0.05
CD36	2	10/10	0.0	>0.05	−3.72 (−5.29, −2.16)	<0.01
(4) Atherosclerosis-preventative mechanisms						
LXRα	4	19/19	22.5	>0.05	2.77 (1.79, 3.76)	<0.01
HO-1	3	30/30	93.9	<0.001	4.20 (0.17, 8.23)	<0.05
SIRT1	3	15/15	56.0	>0.05	3.24 (2.03, 4.46)	<0.01
mTOR	2	7/7	0.0	>0.05	−2.74 (−4.34, −1.15)	<0.01
PCSK9	2	12/12	62.1	>0.05	−5.27 (−7.15, −3.39)	<0.01
NADPH	2	12/12	0.0	>0.05	−6.97 (−9.28, −4.65)	<0.01
NOX2	2	12/12	0.0	>0.05	−1.50 (−2.43, −0.57)	<0.01
p47phox	3	15/15	0.0	>0.05	−5.54 (−7.29, −3.79)	<0.01
P21	2	10/10	84.4	<0.05	−12.68 (−24.36, −1.01)	<0.05

**Table 4:** Subgroup analysis results.

Outcome	Subgroup	□	Studies (*n*)	Heterogeneity	
				*I*^2^ (%)	*p*
Aortic lesion areas	Language Type	English	15	83.7	<0.001
		Chinese	2	59.8	0.115
	Animal species	ApoE^−/−^ mice	15	85.8	<0.001
		C57BL/6 mice	1	-	-
		ApoE*3-Leiden mice	1	-	-
	Animal sex	Male	14	83.4	<0.001
		Female	3	92.5	<0.001
	MT	MT ≤ 8 weeks	3	5.4	0.348
		8 weeks < MT < 16 weeks	7	87.4	<0.001
		MT ≥ 16 weeks	6	45.6	0.102
		Not mentioned	1	-	-
	AT	AT ≤ 8 weeks	4	51.4	0.103
		8 weeks < AT < 16 weeks	7	90.3	<0.001
		AT ≥ 16 weeks	5	53.9	0.07
		Not mentioned	1	-	-
	AM	Intragastric administration	10	80.9	<0.001
		Oral administration	7	71.8	<0.01
	AD	AD ≤ 30 mg/kg	6	84.4	<0.001
		30 mg/kg < AD < 500 mg/kg	5	70.2	<0.01
		AD ≥ 500 mg/kg	6	73.7	<0.01
TC	Language Type	English	14	87.2	<0.001
		Chinese	2	89.5	<0.01
	Animal species	ApoE^−/−^ mice	12	90.8	<0.001
		Wistar rat	2	43.9	0.182
		C57BL/6 mice	1	-	-
		ApoE*3-Leiden mice	1	-	-
	Animal sex	Male	12	87.8	<0.001
		Female	4	93.9	<0.001
	MT	MT ≤ 8 weeks	3	59.0	0.087
		8 weeks < MT < 16 weeks	6	89.9	<0.001
		MT ≥ 16 weeks	6	91.5	<0.001
		Not mentioned	1	-	-
	AT	AT ≤ 8 weeks	2	43.9	0.182
		8 weeks < AT < 16 weeks	8	90.0	<0.001
		AT ≥ 16 weeks	5	93.2	<0.001
		Not mentioned	1	-	-
	AM	Intragastric administration	12	85.3	<0.001
		Oral administration	4	0.0	0.796
	AD	AD ≤ 30 mg/kg	6	76.4	<0.01
		30 mg/kg < AD < 500 mg/kg	6	90.3	<0.01
		AD ≥ 500 mg/kg	4	0.0	0.796
TG	Language Type	English	16	84.4	<0.001
		Chinese	2	29.0	0.235
	Animal species	ApoE^−/−^ mice	14	88.7	<0.001
		Wistar rat	2	72.4	0.057
		C57BL/6 mice	1	-	-
		ApoE*3-Leiden mice	1	-	-
	Animal sex	Male	14	85.9	<0.001
		Female	4	91.3	<0.001
	MT	MT ≤ 8 weeks	5	80.9	<0.001
		8 weeks < MT < 16 weeks	6	48.1	0.086
		MT ≥ 16 weeks	6	91.8	<0.001
		Not mentioned	1	-	-
	AT	AT ≤ 8 weeks	4	66.2	0.031
		8 weeks < AT < 16 weeks	8	85.4	<0.001
		AT ≥ 16 weeks	5	93.2	<0.001
		Not mentioned	1	-	-
	AM	Intragastric administration	12	88.6	<0.001
		Oral administration	6	69.1	<0.01
	AD	AD ≤ 30 mg/kg	6	68.3	<0.01
		30 mg/kg < AD < 500 mg/kg	6	87.0	<0.001
		AD ≥ 500 mg/kg	6	69.1	<0.01
LDL-C	Language Type	English	13	86.1	<0.001
		Chinese	1	-	-
	Animal species	ApoE^−/−^ mice	11	90.5	<0.001
		Wistar rat	2	0.0	0.958
		C57BL/6 mice	1	-	-
	Animal sex	Male	12	87.1	<0.001
		Female	2	96.1	<0.001
	MT	MT ≤ 8 weeks	4	67.6	0.026
		8 weeks < MT < 16 weeks	4	87.5	<0.001
		MT ≥ 16 weeks	6	93.2	<0.001
	AT	AT ≤ 8 weeks	4	67.6	0.026
		8 weeks < AT < 16 weeks	5	83.3	<0.001
		AT ≥ 16 weeks	5	94.0	<0.001
	AM	Intragastric administration	10	87.1	<0.001
		Oral administration	4	86.1	<0.001
	AD	AD ≤ 30 mg/kg	5	84.0	<0.001
		30 mg/kg < AD < 500 mg/kg	5	89.2	<0.001
□	□	AD ≥ 500 mg/kg	4	86.1	<0.001

*Note:* MT, modelling time; AT, administration time; AM, administration method; AD, administered dosage.

## Data Availability

This study did not generate new data.

## Abbreviations

ABCA1, ATP-binding cassette transporter A1; ABCG1, ATP-binding cassette transporter G1; AKT, protein kinase B; AMPK, adenosine-monophosphate-activated protein kinase; AS, atherosclerosis; Bax, Bcl-2-associated X protein; Bcl-2, B-cell lymphoma 2; Caspase, Cysteinyl aspartate specific proteinase; CAT, catalase; CD36, cluster of differentiation 36; CI, confidence intervals; CNKI, China National Knowledge Infrastructure; ECM, extracellular matrix; ECs, endothelial cells; GPX, glutathione peroxidase; HDL-C, high-density lipoprotein cholesterol; HIF1α, hypoxia-inducible factor 1α; HO-1, haem oxygenase 1; ICAM-1, intercellular cell adhesion molecule-1; IL, Interleukin; LDL-C, low-density lipoprotein cholesterol; LDLR, low-density lipoprotein receptor; LXRα, liver X Receptor α; MCP-1, monocyte chemotactic protein-1; MDA, malondialdehyde; mTOR, mechanistic target of rapamycin; NADPH, nicotinamide adenine dinucleotide phosphate; NF-κB, nuclear factor kappa-B; NOX, NADPH oxidases; NRF2, nuclear factor-erythroid 2-related factor 2; oxLDL, oxidized low-density lipoprotein; PCSK9, proprotein convertase subtilisin/kexin type 9; PI3K, phosphatidylinositol-3 kinase; PPARγ, peroxisome proliferator-activated receptor γ; RCT, reverse cholesterol transport; RIS, required information size; ROS, reactive oxygen species; SD, Standard Deviation; SEM, Standard error of mean; SIRT1, sirtuin 1; SMD, standard mean differences; SOD, superoxide dismutase; TC, total cholesterol; TG, triglycerides cholesterol; TGF-β, transforming growth factor-β; TLR4, toll-like receptor 4; TNF-α, tumour necrosis factor-α; TSA, test sequential analysis; VCAM-1, vascular cell adhesion molecule-1; VIP, Chinese Science and Technology Journals Database; VSMCs, vascular smooth muscle cells.
